# Vascular Endothelial Growth Factor and Ki-67 Antigen Expression in Relation to Age and Gender in Oral Squamous Cell Carcinoma

**DOI:** 10.5681/joddd.2012.022

**Published:** 2012-09-01

**Authors:** Noushin Jalayer Naderi, Farrokh Tirgari, Farzin Esmaili, Faranak Paktinat, Zahra Keshavarz

**Affiliations:** ^1^Assistant Professor, Department of Oral and Maxillofacial Pathology, Faculty of Dentistry, Shahed University, Tehran, Iran; ^2^Associate Professor, Cancer Institute, Tehran University of Medical Sciences, Tehran, Iran; ^3^Dentist, Private Practice, Tehran, Iran; ^4^Undergraduate Student, Faculty of Dentistry, Shahed University, Tehran, Iran

**Keywords:** Carcinoma, immunohistochemistry, Ki-67 antigen, VEGF

## Abstract

**Background and aims:**

Vascular endothelial growth factor (VEGF) and Ki-67 antigen are contributing factors in this process cell proliferation and new blood vessels formation in tumor progression. This study was conducted to examine the relationship between the expression of VEGF and Ki-67 and gender and age of patients with oral squamous cell carcinoma (OSCC).

**Materials and methods:**

Twenty-three archival samples of well-differentiated OSCC were examined immunohisto-chemically and assessed by obtaining Total Score (TS = proportion score × staining index). For statistical analysis, t-test and Pearson’s correlation were employed. P≤0.05 was considered statistically significant.

**Results:**

The differences in VEGF expression between males and females (P = 0.43) and different ages (P = 0.88) were not significant. The differences in Ki-67 expression was between males and females (P = 0.67) and different ages (P = 0.88) were also not significant. A positive correlation of VEGF and Ki-67 expression was observed in males and females in addi-tion to ≤ 60 years age group (r = 0.22, r = 0.008, and r = 0.58, respectively; P < 0.05). The expression of VEGF had a nega-tive relation to Ki-67 in > 60 years group (r = −0.48, P < 0.05).

**Conclusion:**

The expression of VEGF and Ki-67 between males and females and different ages were not significant among oral squamous cell carcinoma cases evaluated.

## Introduction

Squamous Cell Carcinoma (SCC) is the most common malignant epithelial tumor of oral cavity.^1^ Oral and oropharyngeal SCC comprises 3% and 2% of total cancers in men and women, respectively. Total mortality rate is 2% for men and 1% for women. The survival rate of patients is 50%.^2^ Oral Squamous Cell Carcinoma (OSCC) is more prevalent in old men. It may manifest with exophytic, endophytic, leukoplakic or erythroplakic appearances and may involve lip and internal structures.^1^

New vessel formation and cell proliferation are essential for tumor growth. Vascular Endothelial Growth Factor (VEGF) is a cytokine that participates in angiogenesis and vascular permeability by inducing the vascular endothelial cell proliferation and migration.^3^ Higher concentrations of VEGF in oral epithelial dysplasia and SCC has been shown before.^4 - 6^

Several studies have tried to demonstrate the angiogenic role of VEGF with regard to prognosis of OSCC.^7 - 9^ In other studies, the association of VEGF expression with histological grade, TNM stage and lymph node metastases have been examined resulting in conflicting data. ^6, 10 - 14^

Cell proliferation is another important factor in tumor progression. Ki-67 antigen is a nuclear protein that is expressed in all cycle cell phases except G0. It has been shown that Ki-67 expression is related to tumor histopathologic grade and prognosis.^15 , 16^

Although different studies have indicated the relationship between VEGF and Ki-67 expression and clinical stage and histopathologic grade of head and neck SCC, the correlation between these two factors and age and gender have not been elucidated in OSCC development. Therefore, the aim of present study was to assess the VEGF and Ki-67 antigen expression in relation to age and gender of patients with OSCC.

## Materials and Methods

In this retrospective study tissue sampling was based on archive. All pathologic records with well-differentiated OSCC diagnosis were retrieved from the archive of Department of Pathology, Cancer Institute, Tehran, Iran. The best fixed samples with sufficient tissue material were selected by examining the hematoxilin-eosin stained slides. Medical records of selected samples were reviewed. In both steps, some samples were excluded because of inadequate or missed tumoral tissue and incomplete medical record information. Finally, 23 formalin-fixed, paraffin embedded samples of well-differentiated OSCC (tongue = 19, floor of the mouth = 2, lower lip = 1, palate = 1) were selected. The inclusion criteria were perfect tissue fixation, adequate tumoral mass for microscopic examination, and absence of necrosis and hemorrhage. Medical records of patients were reviewed and demographic information was registered.

Immunohistochemical Analysis

The biopsies were sectioned at 5 μm thickness and stained with haematoxylin and eosin. These sections were examined by two pathologists. Sections that best coordinated with inclusion criteria were selected. The VEGF and Ki-67 expression were detected immunohistochemically. The 3 μm sections were deparaffinized in xylene, followed by placing in 0.01 M Citrate/HCl Buffer (pH = 6.00) and heated in microwave oven for 10 minutes. After reaching to room temperature, sections were rinsed with phosphate buffered saline (PBS). In the next step, sections were incubated with 1 μg/ml diluted primary antimouse polycolonal and monoclonal antibodies (Dako, Denmark-VEGF and Ki-67 antibody, respectively) for 1 hour and then with biotinylated antibody for 30 minutes. Sections were incubated with peroxidase for 30 minutes and developed in 3,3’diaminobenzidine hydrochloride (DAB). The next step was Mayer’s staining. Before mounting, the sections were immersed in xylene. Between incubations, all samples were rinsed with PBS. The Phaeochromocytoma was used as positive control.

The quantification was completed by light microscopy (Ziess, Japan) at ×400 magnification.

The highest number of positive cells containing sample in each tumor was used for tumor evaluation. VEGF and Ki-67 expression was assessed by obtaining the total score (TS) = proportion score (PS) × staining index (SI), as described by Li et al.^17^ Based on this method, the PS measuring was scored as follows: 0 (no positive tumor cells), 1 (<10% positive tumor cells), 2 (10–50% positive tumor cells), and 3 (>50% positive tumor cells). Staining intensity derived from: 0 (no staining); 1 (weak staining); 2 (moderate staining); and 3 (strong staining). The scoring was blind and was performed twice. Since the mean age was 58.91 years, ‘60’ was considered as age cut off point.

For statistical analyses, t-test and Pearson’s correlation test were employed, using SPSS 13.0 software (SPSS Inc., Chicago, USA). P < 0.05 was considered as statistical significance level.

## Results

There were 13 (56.52%) males and 10 (43.47%) females with the mean age of 58.91 years.

### VEGF Expression

**Figure 1:**
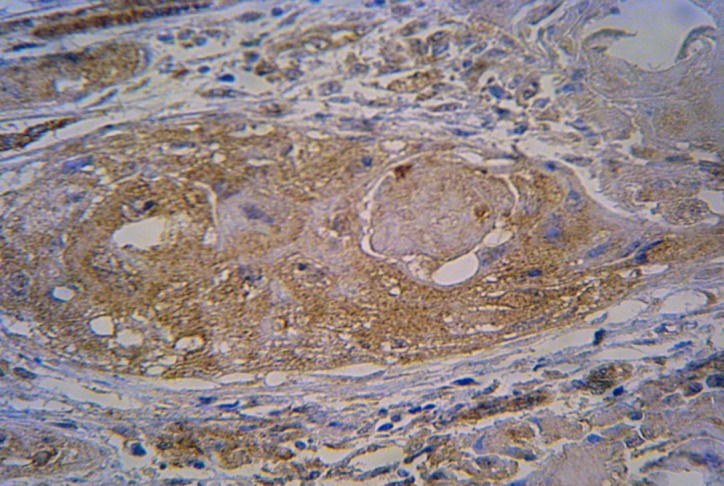
Immunohistochemical staining of VEGF in well-differentiated SCC of tongue (×100)

The positive cells were established by brown-stained of basal and squamous epithelial cells (Figure 1). Endothelial and inflammatory cells, pieces of muscles and red blood cells were also positive in some extents.

The lowest and highest TS were 0 and 9 in females, 0 and 6 in males. Considering the patients’ age, lowest and highest TS were 0 and 9 in ≤ 60 years, 0 and 6 in > 60 years groups. Table 1 shows the TS and mean rank of VEGF expression in relation to gender and age. The differences in VEGF expression between males and females and different ages were not significant (P = 0.43 and P = 0.88, respectively).

**Table 1:** The frequency (mean rank) of vascular endothelial growth factor (VEGF) expression in the evaluated oral squamous cell carcinoma cases according to gender and age group

	Total score									
Factor	0	1	2	3	4	5	6	7	8	9
Gender										
Female	2 (14.5)	0 (1)	1 (12)	3 (16.5)	0 (6)	0 (6)	3 (16.5)	0 (6)	0 (6)	1 (12)
Male	3 (16.5)	3 (16.5)	1 (12)	4 (18)	0 (6)	0 (6)	2 (14.5)	0 (6)	0 (6)	0 (6)
Age group										
<=60	2 (14)	2 (14)	2 (14)	4 (18.5)	0 (1)	0 (5)	1 (11)	0 (5)	0 (5)	1 (11)
≤60	3 (16.5)	1 (11)	0 (5)	3 (16.5)	0 (5)	0 (5)	4 (18.5)	0 (5)	0 (5)	0 (5)
Mean age years	64	56.7	61.5	52.7	0	0	68.4	0	0	31

Total score calculated as proportion score × staining index

### Ki-67 Expression

**Figure 2:**
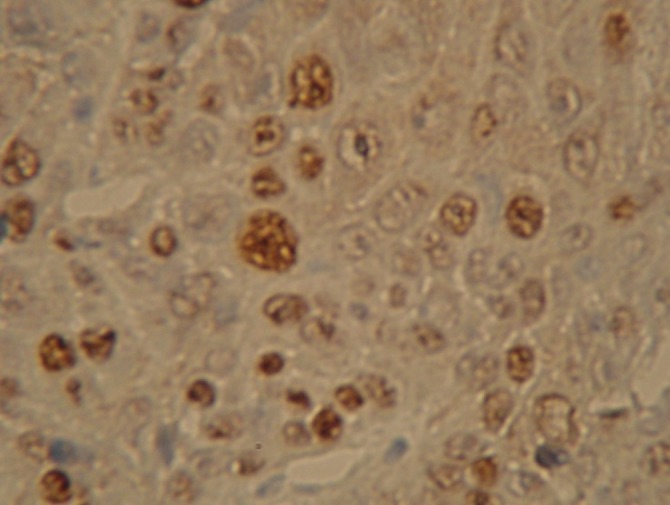
Immunohistochemical staining of Ki-67 in well-differentiated SCC of tongue (×400)

Light to dark brown color of nuclei were considered positive for Ki-67 expression (Figure 2). Ki-67 positive cells were also observed in nerve, muscle and salivary gland tissues. Lowest and highest TS were 1 and 9 in females, 1 and 6 in males. Considering the patients age, lowest and highest TS were 1 and 9 in both ≤ 60 years and > 60 years groups.

Table 2 shows the TS and mean rank of Ki-67 expression in relation to gender and age.

**Table 2:** The frequency (mean rank) of Ki-67 expression in the evaluated oral squamous cell carcinoma cases according to gender and age group

	Total score								
Factor	1	2	3	4	5	6	7	8	9
Gender									
Female	1 (10)	0 (1)	2 (13.5)	3 (16.5)	0 (4.5)	1 (10)	0 (4.5)	0 (4.5)	3 (16.5)
Male	2 (13.5)	1 (10)	2 (13.5)	2 (13.5)	0 (4.5)	6 (18)	0 (4.5)	0 (4.5)	0 (4.5)
Age									
≤60	2 (13.5)	0 (1)	1 (10.5)	5 (18)	0 (4.5)	3 (15.5)	0 (4.5)	0 (4.5)	1 (10.5)
>60	1 (10.5)	1 (10.5)	3 (15.5)	0 (4.5)	0 (4.5)	4 (17)	0 (4.5)	0 (4.5)	2 (13.5)
Mean age years	56	64	65.3	51.6	0	58.4	0	0	65

Total score calculated as proportion score × staining index

The differences in Ki-67 expression between males and females and different ages were not significant (P = 0.67 and P = 0.88, respectively).

A positive correlation of VEGF and Ki-67 expression was observed in males and females in addition to ≤ 60 years age group (r = 0.22, r = 0.008, and r = 0.58, respectively; P < 0.05). The expression of VEGF had a negative relation to Ki-67 in > 60 years group (r = −0.48, P < 0.05).

## Discussion

The results of this study show that the expression of VEGF and Ki-67 between males and females and different ages are not significant in well-differentiated OSCC. This finding is in agreement with Sun et al,^22^ demonstrating the statistically insignificant VEGF and Ki-67 expression in laryngeal SCC at different ages and genders.

Consistent with a number of studies, our study indicated a positive expression of Ki-67 in SCC.^18 , 19^ Ki-67 over-expression has been reported from epithelial dysplasia to SCC. ^20, 21^ These results indicate the importance of Ki-67 expression in biologic outcome of the tumor.

Since most studies have focused on the results of VEGF and Ki-67expression in relation to tumor stage and lymph node metastasis, studies about the importance of age and gender on tumor progression are very few. To the best of our knowledge, this is the first study in this regard, considering age and gender factors.

Is there any correlation between angiogenesis and cell proliferation and tumor progression? Bourlev et al^23^ have reported high proliferative activity accompanied by higher local angiogenic activity in endometriosis and endometriotic lesions. In paragangliomas, Brieger et al^24^ have demonstrated higher Ki-67 counts in VEGF positive tumors.

On the other hand, Mineta et al^6^ have reported that VEGF over-expression was not correlated with Ki-67 and P53 over-expression in tongue SCC. Sun et al^25^ have reported a positive correlation between Ki-67 and VEGF expression in laryngeal SCC. A positive correlation between VEGF and Ki-67 expression and males and females and ≤ 60 years group was observed in this study, but the expression of VEGF had a negative relation to Ki-67 in > 60 years.

The reason for such conflicting findings can not be explained by means of current knowledge, and therefore, further investigations are necessary.

Although Ki-67 expression is a definite indicator of cell proliferation, the VEGF expression has been shown in normal, hyperplastic and cancerous squamous lesions.^13 , 14^ VEGF expression has also been demonstrated in embryo and human organs. Considering these facts, it seems that the role of VEGF in tumor progression is independent. Tae et al^13^ suggested that VEGF regulates mucosa function under physiologic conditions.

Taking together, it is possible to conclude that angiogenesis and cell proliferation are simultaneous events which promote tumor progression with separated mechanisms.

It seems that the quality of angiogenesis and cell proliferation are the same in males and females and different ages. These details reveal that the outcome of OSCC is multifactorial and patients’ age and gender are cofactors in this regard. In this study, the interaction of age and gender was not determined, and therefore, further studies on the interaction of contributing factors in tumor progression are warranted.
